# The lipid-sensor TREM2 aggravates disease in a model of LCMV-induced hepatitis

**DOI:** 10.1038/s41598-017-10637-y

**Published:** 2017-09-12

**Authors:** Lindsay Kosack, Riem Gawish, Alexander Lercher, Bojan Vilagos, Anastasiya Hladik, Karin Lakovits, Anannya Bhattacharya, Christopher Schliehe, Ildiko Mesteri, Sylvia Knapp, Andreas Bergthaler

**Affiliations:** 1 CeMM Research Center for Molecular Medicine of the Austrian Academy of Sciences, Lazarettgasse 14 AKH BT25.3, 1090 Vienna, Austria; 2 Department of Medicine I, Laboratory of Infection Biology, Medical University of Vienna, 1090 Vienna, Austria; 3 Department of Biomedical Science, Institute of Animal Breeding and Genetics, University of Veterinary Medicine Vienna, 1210 Vienna, Austria; 4 Institute of Pathology Überlingen, 88662 Überlingen, Germany; 5 Present Address: Department of Immunology, St. Jude Children’s Research Hospital, Memphis, TN 38105 USA; 6 Present Address: Department of Immunology, Erasmus MC, University Medical Center Rotterdam, 3015CN Rotterdam, The Netherlands

## Abstract

Lipid metabolism is increasingly being appreciated to affect immunoregulation, inflammation and pathology. In this study we found that mice infected with lymphocytic choriomeningitis virus (LCMV) exhibit global perturbations of circulating serum lipids. Mice lacking the lipid-sensing surface receptor triggering receptor expressed on myeloid cells 2 (*Trem2* ^−/−^) were protected from LCMV-induced hepatitis and showed improved virus control despite comparable virus-specific T cell responses. Non-hematopoietic expression of TREM2 was found to be responsible for aggravated hepatitis, indicating a novel role for TREM2 in the non-myeloid compartment. These results suggest a link between virus-perturbed lipids and TREM2 that modulates liver pathogenesis upon viral infection. Targeted interventions of this immunoregulatory axis may ameliorate tissue pathology in hepatitis.

## Introduction

Immunoregulatory processes are crucial to maintain effective pathogen control while preventing excessive inflammation and immunopathology^1–3^. Next to epigenetic, transcriptional and post-transcriptional mechanisms, the crosstalk between inflammatory and metabolic processes provides an additional layer of regulation^4, 5^. Lipids and their interactions with the immune system have received particular attention^6–14^ and have been implicated in inflammatory diseases such as metabolic disorders, atherosclerosis and rheumatoid arthritis^15–17^. A central hub of lipid metabolism is the liver, which also provides a niche for viruses like hepatitis B virus (HBV) and hepatitis C virus (HCV)^18, 19^. Interestingly, lipids play a key role at the different stages of the viral life cycle including cell entry, replication and budding^20–22^. In turn, viral infections may result in alterations in the extracellular composition of lipid species, which are thought to influence inflammatory processes and disease progression^23–26^. This is exemplified by HBV and HCV, which rely on host lipid metabolism to establish a successful infection and at the same time induce changes in serum lipids in patients^22, 25, 27–31^. The precise mechanisms of how the host reacts to such changes and its impact on tissue pathology still remain to be fully explored.

Several lipid-sensors such as the G-protein coupled receptors sphingosine-1-phosphate receptors (S1PR1-S1PR5) and prostaglandin receptors (e.g. EP2 and EP4) are already known to be involved in immunoregulation and antiviral immune responses^10, 32, 33^. Recently, the surface receptor triggering receptor expressed on myeloid cells 2 (TREM2), which previously was reported to bind anionic ligands and apolipoprotein E (ApoE)^34–37^, was shown to sense glycerophospholipids and sphingomyelins^38, 39^. TREM2 is a member of the immunoglobulin (Ig) superfamily and is expressed on many cell populations including Kupffer cells, microglia, alveolar macrophages, bronchial epithelial cells and peritoneal macrophages^40–44^. It signals through the adaptor protein DNAX activation protein 12 (DAP12), which results in downstream signal transduction events including the phosphorylation of phospholipase (PL) Cγ and extracellular signal-related kinase (ERK) 1/2 as well as the modulation of peroxisome proliferator-activated receptor-δ (PPAR-δ) activity^43, 45, 46^. In addition, DAP12 is associated with numerous other cell surface receptors having either activating or inhibitory downstream effects on immunoregulation^47, 48^.

TREM2 deficiency in macrophages, dendritic cells and microglia leads to increased production of pro-inflammatory cytokines upon stimulation with toll-like receptor ligands^49–52^. Likewise, *Trem2* ^−/−^ bone marrow-derived macrophages and peritoneal macrophages produce increased levels of TNFα and IL-6 upon bacterial stimulation^43, 53^. Yet, there is evidence that TREM2 deficiencies can also attenuate inflammatory responses in macrophages and dendritic cells^43, 54, 55^ as well as in a brain injury model^56^, indicating that the function of TREM2 may vary in a context-dependent manner. Loss-of-function mutations in the human *TREM2* gene have been linked to the fatal Nasu-Hakola disease, which is associated with bone fractures, progressive dementia and epileptic seizures^57, 58^. Recent genome-wide association studies identified the missense mutation R47H in *TREM2* to confer an increased risk for Alzheimer’s disease (AD)^38, 59, 60^. These insights instigated increased interest in the biology of TREM2 with a primary focus on the central nervous system and bone tissue.

In this study we employed the model of lymphocytic choriomeningitis virus (LCMV), which represents a benchmark model of acute and chronic viral infection and results in hepatitis as a consequence of immunopathological mechanisms^61–64^. We found that infection with LCMV perturbs the composition of circulating lipid species in wild type (WT) mice, which prompted us to investigate the role of the recently described lipid-sensing receptor TREM2. By using *Trem2* ^−/−^ mice we could identify a novel disease-aggravating role of non-hematopoietically-expressed TREM2 in virus-induced liver pathology.

## Results

### Viral infection perturbs the serum composition of metabolites

**Figure 1:**
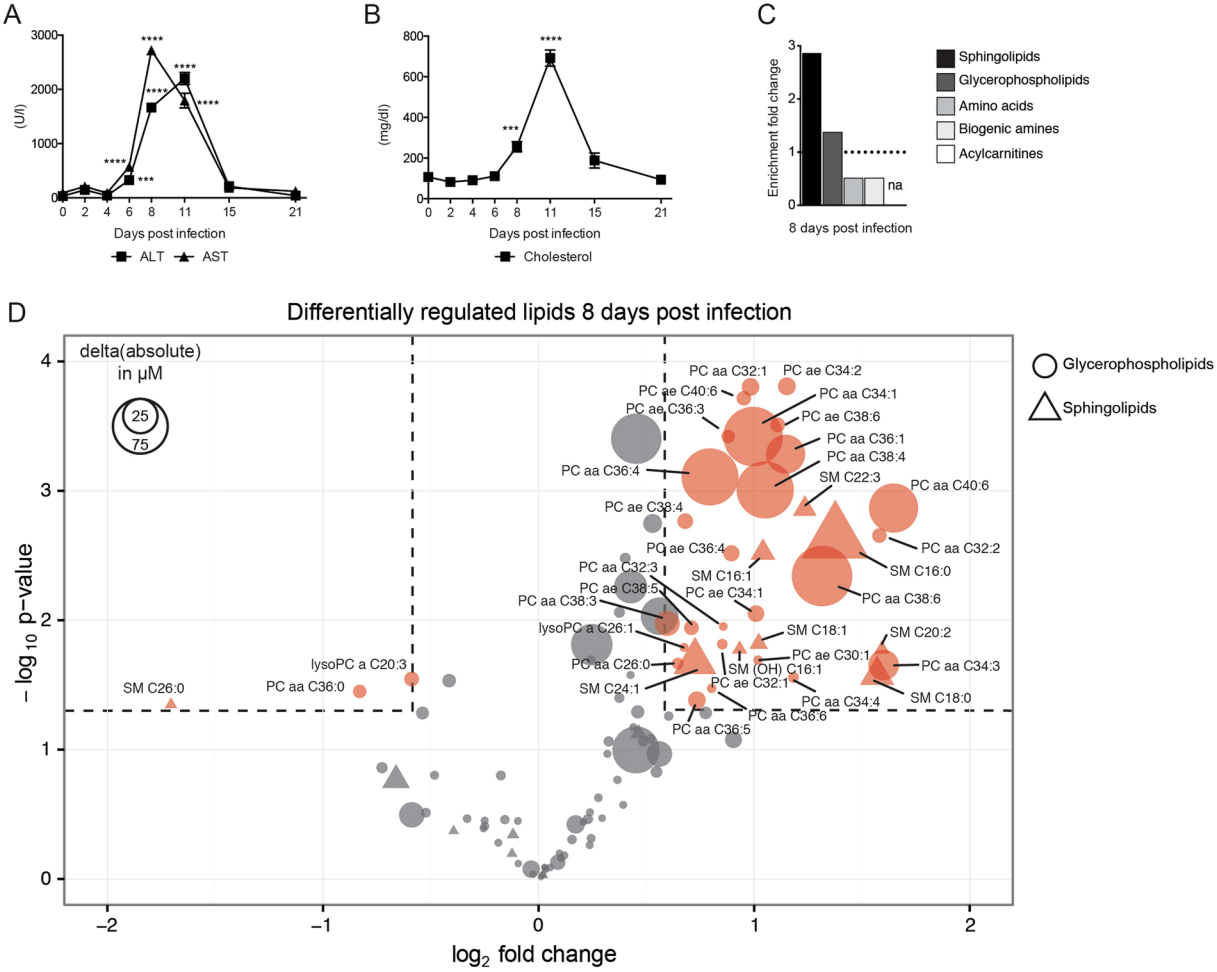
LCMV infection induces global changes in the composition of serum metabolites. WT mice were infected with LCMV strain WE, and serum was collected at various time-points post infection (**A**–**D**). Serum kinetics of (**A**) alanine aminotransferase (ALT) and aspartate aminotransferase (AST), and (**B**) cholesterol (n = 5 mice per group). (**A**,**B**) Statistical significance was calculated by One-way ANOVA with Bonferroni correction comparing the different time points to day 0. Symbols in (**A**,**B**) represent the mean ± SEM. For targeted metabolomics (**C**,**D**), serum from uninfected mice and mice that were infected with LCMV 8 days previously was used (n = 4 mice per group). (**C**) Fold enrichment of the metabolite classes (glycerophospolipids, sphingolipids, biogenic amines, amino acids, acylcarnitines) was calculated using the relative proportion (number of significant metabolites from a specific metabolite class divided by the number of all significant metabolites measured) normalized to the number of measured metabolites of a specific metabolite class divided by the number of all measured metabolites. Members of the acylcarnitine class did not pass the threshold of fold-change >1.5 and p-value < 0.05. (**D**) Bubble plot of individual glycerophospholipids (circle) and sphingolipids (triangle) depicting fold-change (x-axis), p-value (y-axis) and the absolute change (size of symbols). Statistical significance of changed individual metabolites in sera of infected vs. uninfected mice was calculated with unpaired t-test (**C**,**D**). Red symbols in (**D**) reflect metabolites with a fold-change >1.5 and a p-value < 0.05.

To investigate the changes in serum metabolites after virus infection, we infected WT mice with LCMV strain WE^61, 63^. Infected mice exhibited elevated serum concentrations of the clinical hallmark parameters of hepatitis, alanine aminotransferase (ALT) and aspartate aminotransferase (AST) (Fig. 1A) and elevated serum concentrations of cholesterol (Fig. 1B). Next, we wanted to assess systemic changes of serum metabolites upon viral infection by a targeted metabolomics approach. We infected WT mice with LCMV and collected sera before and during the peak of hepatitis. Quantification of 188 metabolites was performed by mass-spectrometry unveiling an enrichment of altered glycerophospholipid and sphingolipid species in the serum of infected mice on day 8 post infection (Fig. 1C, Table S1). Other metabolites such as acylcarnitines, amino acids and biogenic amines showed less drastic changes (Fig. 1C, Table S1). Of the quantified 91 different glycerophospholipids and 15 sphingolipids, we observed a complex pattern of up- and downregulated individual lipid species at both two and eight days post infection (Fig. 1D, Fig. S1A). These lipids are a class of metabolites with intricate regulatory mechanisms and multiple known roles including the involvement in hepatocellular death and liver diseases^65–67^. Together, this data shows that LCMV infection perturbs the global serum composition of lipids including the upregulation of numerous species of glycerophospholipids and sphingolipids.

### Lack of TREM2 ameliorates liver damage upon viral infection

TREM2 was recently revealed to be a lipid-sensing receptor that binds glycerophospholipids and sphingomyelin^38^. Indeed, addition of lipids extracted from sera of infected mice tended to increase the activity of Ppar-δ in WT but not in *Trem2* ^−/−^ cells (Fig. S1B)^43^. Similarly, individual lipid species, which were significantly upregulated in the sera of mice infected eight days previously, led to increased reporter activation in a TREM2-dependent manner (Fig. S1C).

**Figure 2:**
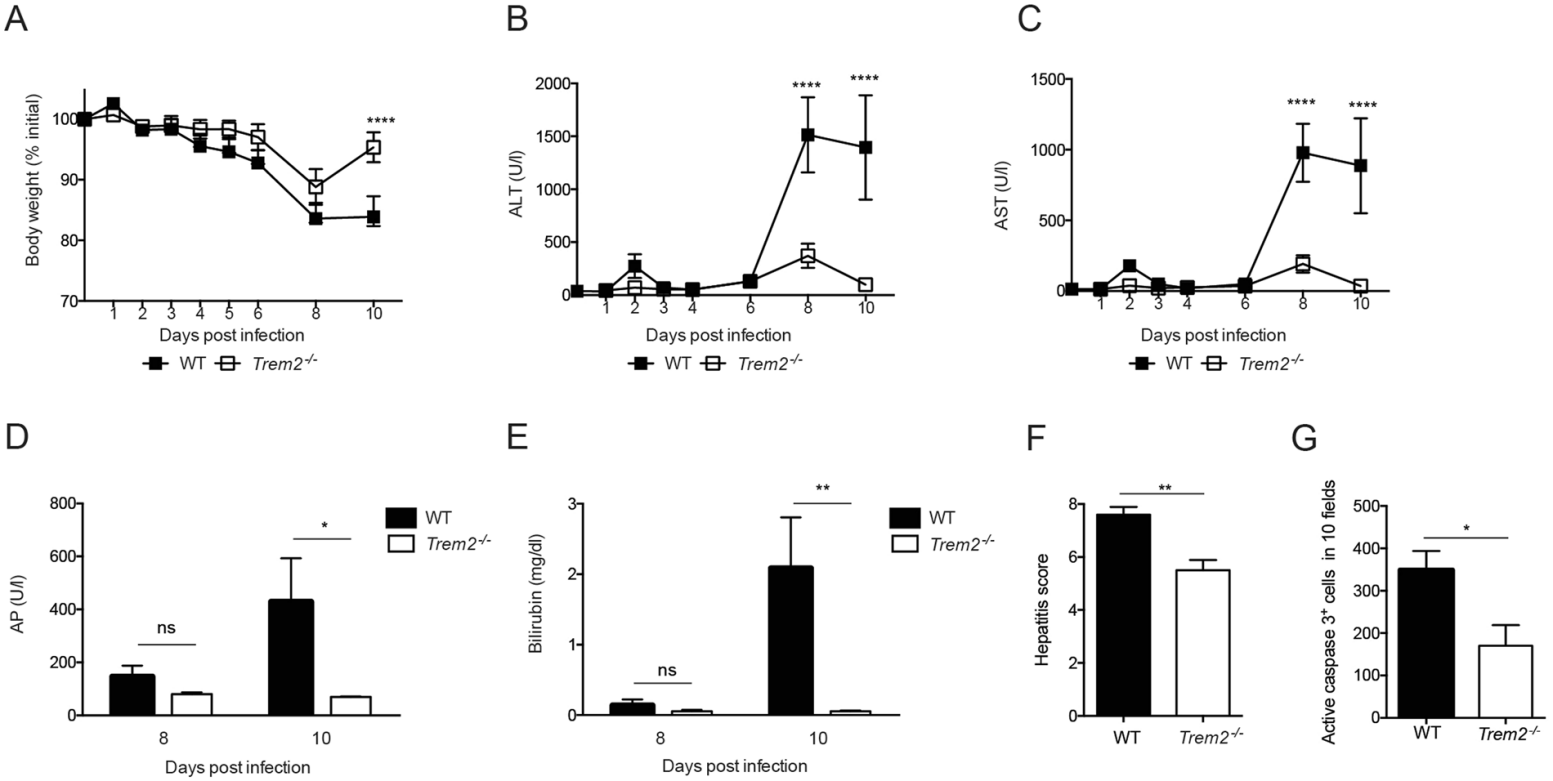
*Trem2* ^−/−^ mice are protected against LCMV-induced hepatitis. WT and *Trem2* ^−/−^ mice were infected with LCMV strain WE. (**A**) Body weight was monitored after LCMV infection (n = 5 mice per group, representative of ≥ two independent experiments). (**B**,**C**) Serum kinetics of alanine aminotransferase (ALT) (**B**) and aspartate aminotransferase (AST) (**C**) were measured after LCMV infection (n = 5 mice per group, representative of ≥ two independent experiments). (**D**,**E**) Serum levels of alkaline phosphatase (AP) (**D**) and bilirubin (**E**) were measured eight and ten days post infection (n = 5 mice per group, representative of two independent experiments). (**F**) Liver sections of infected WT and *Trem2* ^−/−^ were histopathologically scored (see Methods; n = 5 mice per group). (**G**) Liver sections of infected WT and *Trem2* ^−/−^ were stained for cleaved Caspase 3 and the number of positive cells in 10 fields was quantified (n = 4 mice per group). Statistical significance was calculated by (**A**–**E**) Two-way ANOVA with Bonferroni correction or by (**F**,**G**) unpaired t-test. Symbols respectively bars represent the mean ± SEM.

After observing differential regulation of systemic lipid species after LCMV infection and their effects on a TREM2-dependent reporter system, we hypothesized a potential role for TREM2 in LCMV hepatitis. We infected *Trem2* ^−/−^ and WT mice and observed that mice lacking TREM2 lost less body weight compared to infected WT mice (Fig. 2A). This was associated with reduced serum concentrations of ALT and AST in infected *Trem2* ^−/−^ compared to WT mice (Fig. 2B,C). Moreover, *Trem2* ^−/−^ mice exhibited lower serum concentrations of two alternative parameters for liver damage, alkaline phosphatase (AP) and bilirubin, compared to WT mice upon viral infection (Fig. 2D,E). In line with these findings, a histological assessment of liver damage revealed a lower pathological score (Fig. 2F) and fewer cleaved Caspase 3^+^ cells (Fig. 2G) in infected *Trem2* ^−/−^ mice compared to WT mice. Yet, serum concentrations of the kidney-related parameters blood urea nitrogen and creatinine were comparable between infected WT and *Trem2* ^−/−^ mice (Fig. S2A,B), suggesting that the differences in tissue damage were not generalized to all organs. We also performed a metabolic profiling of *Trem2* ^−/−^ mice on day 8 post infection and found significant changes in the lipid profiles compared to WT mice, likely reflecting the extent of pathology (Table S1). In summary, the lack of TREM2 resulted in an unexpected amelioration of LCMV-induced liver damage.

### *Trem2*^−/−^ mice show improved virus clearance

**Figure 3:**
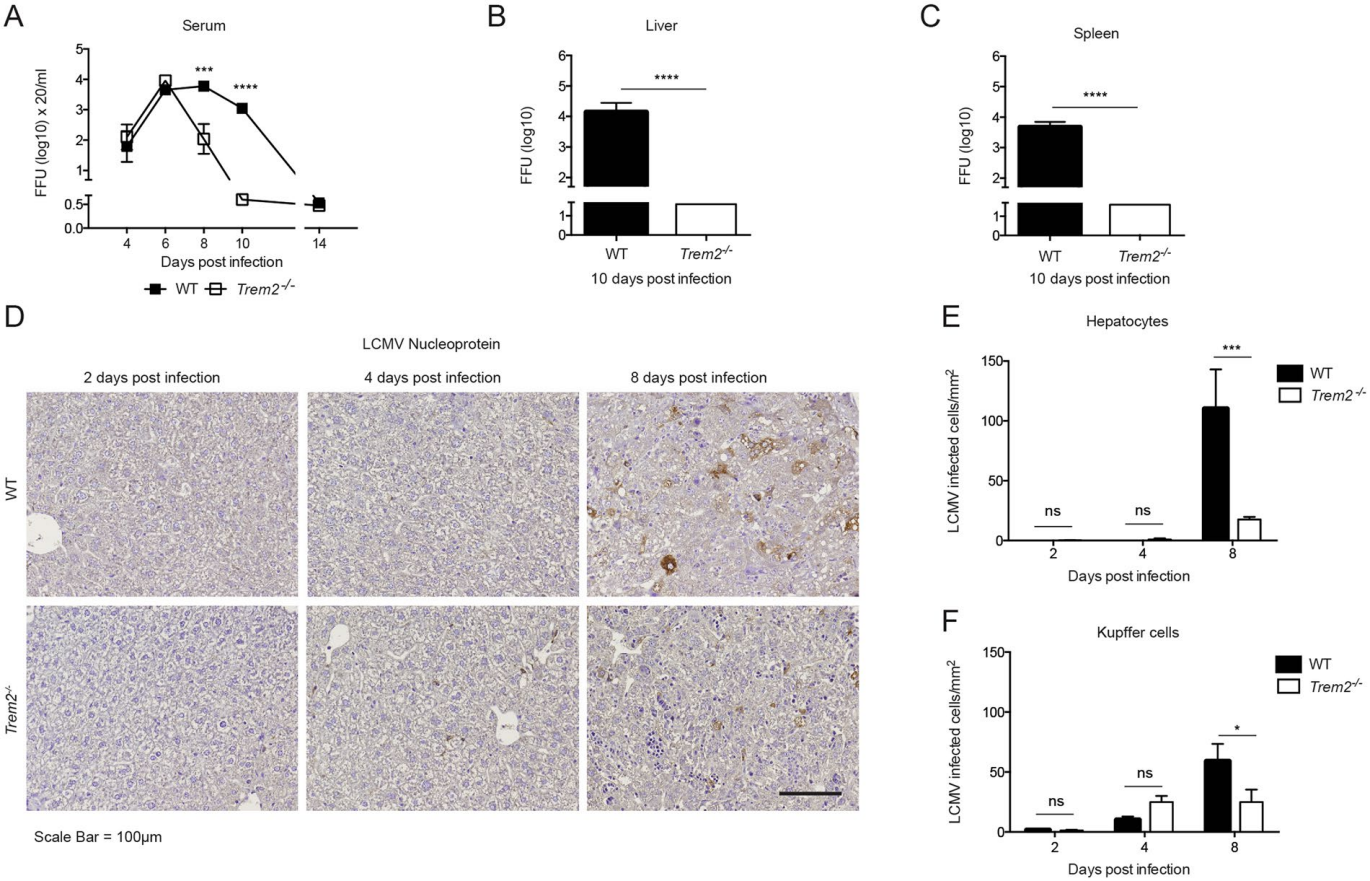
*Trem2* ^−/−^ mice show improved virus clearance compared to WT mice. WT and *Trem2* ^−/−^ mice were infected with LCMV strain WE. (**A**–**C**) Viral loads from serum (**A**), liver (**B**) and spleen (**C**) were quantified by immunological focus assay (n = 4–5 mice per group, representative of two independent experiments (**B**,**C**), data pooled from two independent experiments (**A**). (**D**,**F**) Liver sections from infected WT and *Trem2* ^−/−^ mice were stained for LCMV nucleoprotein (NP). Representative images of 3 to 4 mice per condition are shown in (**D**) and NP^+^ hepatocytes and NP^+^ Kupffer cells were quantified (**E**,**F**). Scale bar represents 100 μM. Statistical significance was calculated by (**A**,**E**,**F**) Two-way ANOVA with Bonferroni correction or by (**B**,**C**) unpaired t-test. Symbols respectively bars represent the mean ± SEM.

To investigate viral propagation in WT and *Trem2* ^−/−^ mice, we determined the infectious viral loads by focus-forming assay (Fig. 3A–C). Both genotypes had comparable viral loads in the serum until day six (Fig. 3A). At later time points, however, *Trem2* ^−/−^ mice exhibited reduced viral loads in serum (Fig. 3A), liver (Fig. 3B) and spleen (Fig. 3C) compared to WT mice. These findings were corroborated by immunohistochemical staining of liver sections, whereby reduced amounts of the viral nucleoprotein (NP) in *Trem2* ^−/−^ mice compared to WT mice were detected at 8 days post infection (Fig. 3D). Hepatocytes and Kupffer cells are the most abundant cell types in the liver and represent the primary target of LCMV in this organ^61^. We thus quantified the signal of viral NP in these cell populations and detected reduced numbers of NP^+^ hepatocytes and NP^+^ Kupffer cells in *Trem2* ^−/−^ liver sections compared to WT mice (Fig. 3E,F). The lack of the cellular receptor TREM2 *per se* may affect viral entry or replication. We therefore infected primary bone marrow-derived macrophages (BMDMs) from both genotypes but found similar viral growth curves in WT and *Trem2* ^−/−^ BMDMs (Fig. S3). In summary, *Trem2* ^−/−^ mice show improved viral clearance compared to WT mice.

### WT and *Trem2*^−/−^ mice mount comparable T cell responses

**Figure 4:**
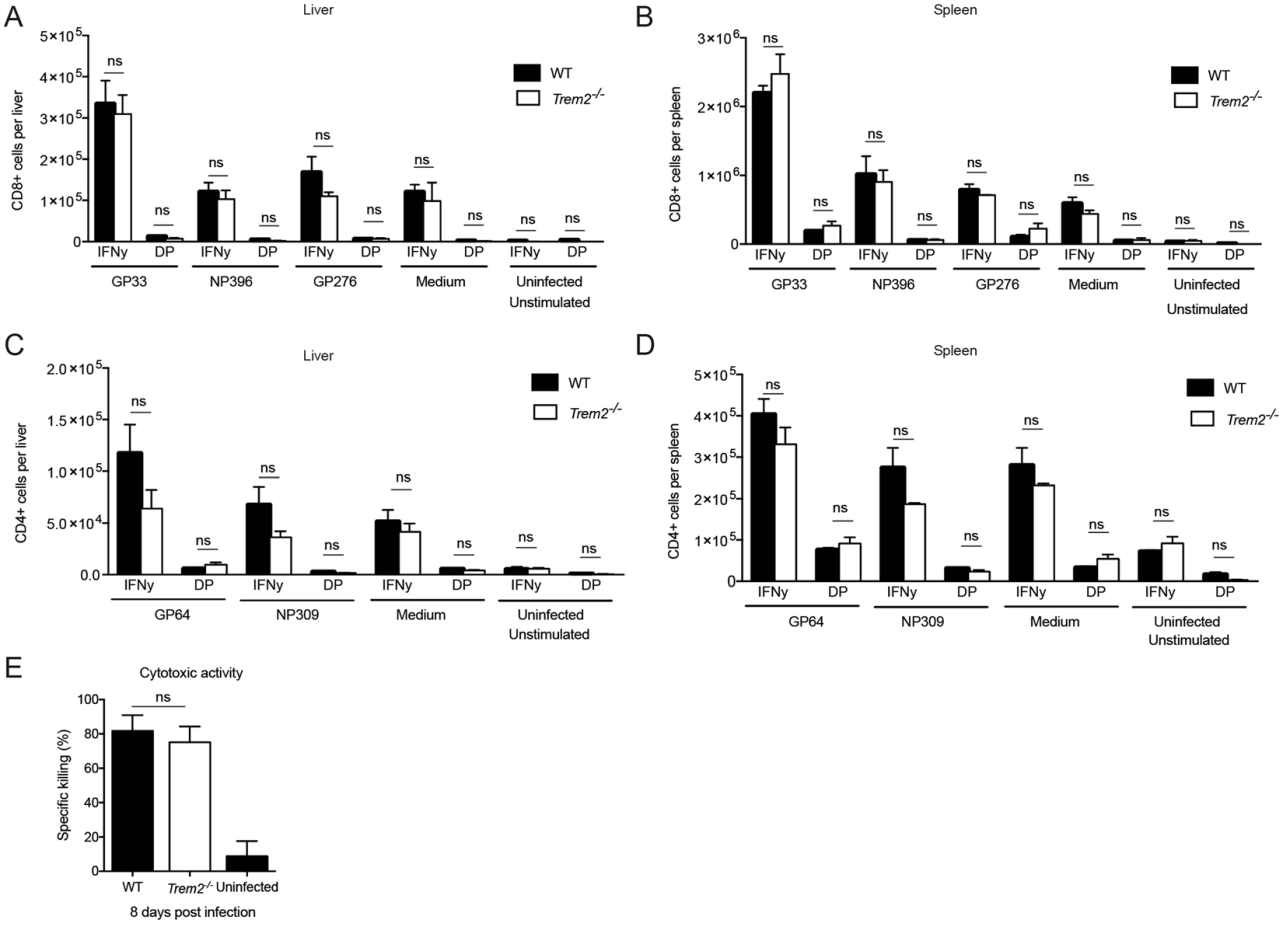
WT and *Trem2* ^−/−^ mice show indistinguishable T cell responses. WT and *Trem2* ^−/−^ mice were infected with LCMV strain WE. (**A**,**B**) Single cell suspensions of liver and spleen were restimulated *ex vivo* with peptides GP_33–41_, NP_396–404_ and GP_276–286_. CD8+ T cells producing IFNγ and double-producing (DP) IFNγ and TNFα were quantified in the liver (**A**) and spleen (**B**) eight days post infection (n = 3 mice per group). (**C**,**D**) Single cell suspensions of liver and spleen were restimulated *ex vivo* with peptides GP_64–80_ and NP_309–328_. CD4+ T cells producing IFNγ and double-producing IFNγ and TNFα were quantified in the liver (**C**) and spleen (**D**) eight days post infection (n = 3 mice per group). Statistical significance was calculated by unpaired t-test. Bars represent the mean ± SEM. (**E**) Eight days post infection, virus-specific cytotoxic activity of CD8^+^ T cells from WT and *Trem2* ^−/−^ mice was measured using an *in vivo* cytotoxicity assay (n = 3–4 mice per group). Statistical significance was calculated by unpaired t-test. Bars represent the mean ± SEM.

Virus control in the LCMV model is dependent on T cells and the damage observed in LCMV-induced hepatitis is largely attributed to CD8 T cells^63^. To assess T cell responses in our model, we infected WT and *Trem2* ^−/−^ mice with LCMV and enumerated CD8^+^ effector memory (EM) and CD4^+^ EM T cells as well as viral antigen-specific CD8 and CD4 T cell responses from spleen and liver tissue by intracellular cytokine staining using flow cytometry (Fig. 4, Fig. S4). Infected WT and *Trem2* ^−/−^ mice had comparable percentag﻿e﻿ of﻿ E﻿M﻿ T cells (Fig. S4), an﻿﻿d indistinguishable﻿ CD8 T cell numbers of IFNγ single producers and IFNγ TNFα double producers upon peptide stimulation for the viral epitopes GP_33–41_, NP_396–404_ and GP_276–286_ in both liver and spleen (Fig. 4A,B). This experiment indicated that WT and *Trem2* ^−/−^ mice mount similar CD8 T cell responses upon viral infection. Likewise, we assessed the cytokine producing-capacity of virus-specific CD4 T cells from liver and spleen. To that end, we stimulated cells with peptides for the CD4 T cell epitopes GP_61–80_ and NP_309–328_ and analyzed IFNγ single producers and IFNγ TNFα double producers (Fig. 4C,D). In analogy to the CD8 T cell results, this analysis revealed comparable CD4 T cell responses in infected WT and *Trem2* ^−/−^ mice. In addition, CD8^+^ T cells from both WT and *Trem2* ^−/−^ mice were equally able to lyse target cells loaded with the viral epitope peptide GP_33–41_ *in vivo* (Fig. 4E), indicating comparable CD8 T cell-mediated cytotoxicity. In line with this, WT and *Trem2* ^−/−^ mice exhibited comparable elevation of serum transaminases upon injection of concanavalin A (Fig. S5), a model of acute hepatitis which depends on generalized antigen-independent activation and recruitment of T cells to the liver^68^.

Together, we found that LCMV-infected WT and *Trem2* ^−/−^ mice mount comparable virus-specific CD8 and CD4 T cell responses in spleen and liver, suggesting that the observed differences in liver pathology and virus clearance are unlikely to be driven by T cell proliferation, migration, cytokine production or cytotoxicity.

We also profiled γ/δ T cells, NK cells, NKT cells, neutrophils and macrophages in spleen and liver of WT and *Trem2* ^−/−^ mice upon infection by flow cytometry (Fig. S6). This analysis revealed largely comparable numbers of cell populations albeit we noticed an increase of NK and NKT cells in *Trem2* ^−/−^ mice compared to WT mice on day 4 post infection (Fig. S6B,C,G,H).

### Comparable cytokine responses in WT and *Trem2*^−/−^ mice upon LCMV infection

In order to investigate the induction of cytokines before the peak of LCMV-induced hepatitis, we quantified protein concentrations of chemokine (C-X-C motif) ligand 1 (CXCL1), interleukin 6 (IL-6) and interferon alpha (IFNα) in sera of WT and *Trem2* ^−/−^ mice by ELISA (Fig. S7). This revealed comparable levels of IL-6 and CXCL1 in WT and *Trem2* ^−/−^. Yet, *Trem2* ^−/−^ mice exhibited reduced levels of IFNα compared to WT mice upon LCMV infection.

### Non-hematopoietic TREM2 aggravates LCMV induced hepatitis

**Figure 5:**
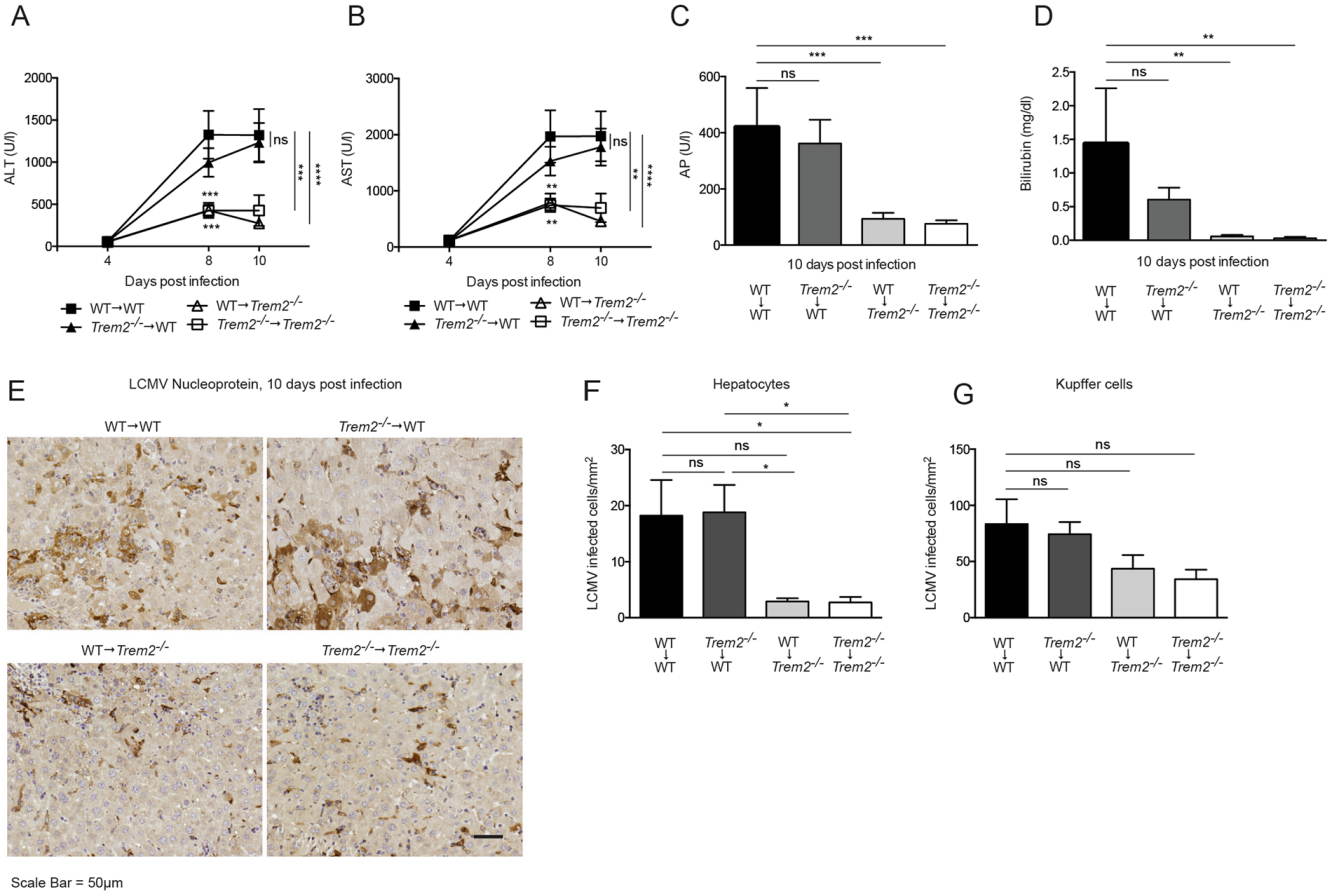
Non-hematopoietic TREM2 aggravates LCMV-induced hepatitis. (**A**–**G**) Chimeric mice were generated by reciprocal transfer of WT and *Trem2* ^−/−^ bone marrow and subsequently infected with LCMV strain WE. (**A**,**B**) Serum kinetics of alanine aminotransferase (ALT) (**A**) and aspartate aminotransferase (AST) (**B**) were measured after LCMV infection (n = 8–9 mice per group, data pooled from two similar experiments). (**C**,**D**) Serum levels of alkaline phosphatase (AP) (**C**) and bilirubin (**D**) were measured ten days post infection (n = 8–9 mice per group, data pooled from two similar experiments). (**E**–**G**) Liver sections from infected mice were stained for LCMV nucleoprotein. Representative images are shown in (**E**) and LCMV^+^ hepatocytes and Kupffer cells were quantified (**F**,**G**) (n = 8–9 mice per group, data pooled from two similar experiments harvested at 10 respectively 12 days post infection). Scale bar represents 50 μM. Statistical significance was calculated by (**A–D**) Two-way or by (**F**,**G**) One-way ANOVA with Bonferroni correction. Symbols respectively bars represent the mean ± SEM.

To determine the cellular compartment relevant for the observed TREM2 dependent liver pathology, we performed a reciprocal bone marrow transfer (Fig. 5). As a subset of Kupffer cells is radio-resistant^69^, we additionally treated the chimeric mice with liposomal clodronate to ablate remaining Kupffer cells in irradiated mice as demonstrated previously^62, 69^. Reconstitution of bone marrow was confirmed by flow cytometry (Fig. S8). Subsequently, the four groups of chimeric mice were infected with LCMV and serum concentrations of ALT, AST, AP and bilirubin were determined (Fig. 5A–D). These results revealed reduced liver damage in WT - > *Trem2* ^−/−^ and *Trem2* ^−/−^ - > *Trem2* ^−/−^ chimeric mice, consistent with a loss of TREM2 in radio-resistant cells. In line with these results, chimeric mice lacking non-hematopoietically expressed TREM2 showed reduced viral loads in hepatocytes (Fig. 5E–G). TREM2 is expressed in macrophages but not at detectable levels in hepatocytes (Fig. S9A,B), which may suggest the involvement of another non-hematopoietic cell population. Collectively, these experiments show that loss of TREM2 in the non-hematopoietic compartment results in ameliorated viral hepatitis.

## Discussion

In this study, we report a novel pathogenic role for TREM2 that results in aggravated viral hepatitis. Mice lacking the lipid-sensor TREM2 and WT mice were analyzed using the model of LCMV-induced hepatitis, which is largely T-cell dependent^63^. Interestingly, we found reduced liver pathology in *Trem2* ^−/−^ mice compared to WT mice despite comparable virus-specific T cell responses. This may be due to accelerated virus clearance in the *Trem2* ^−/−^ mice and reduced virus spread from Kupffer cells to hepatocytes between day 4 and 8 post infection^61^. Of note, we found increased levels of NK and NKT cells in *Trem2* ^−/−^ mice compared to WT mice preceding the onset of liver pathology. This could indicate an involvement of these cell populations, which have been implicated in the control of LCMV^70, 71^. We also detected reduced levels of serum IFNα in *Trem2* ^−/−^ mice upon infection. It is tempting to speculate that IFNα which is known to impact not only the antiviral cellular state but also the interactions between NK and T cells^72^, could provide additional cues for the underlying molecular mechanisms that govern the observed virus-induced TREM2-dependent phenotype.

TREM2 appears to exert highly specific effects in a cell-type specific and possibly pathogen-specific manner. The discovery that TREM2 is involved in Alzheimer’s disease led to a strong focus on its role in the central nervous system (CNS)^59, 60^. However, many other studies suggest that TREM2, in addition to its important role in the CNS, plays an immunomodulatory role during infection. For example, TREM2 has been shown to promote the clearance of *Pseudomonas aeruginosa* keratitis^73^ and injection of bone marrow cells over-expressing TREM2 improved bacterial phagocytosis in a model of cecal ligation and puncture^74^. In contrast, lack of TREM2 did not impact the outcome of bacterial peritonitis with *Escherichia coli* ^53^ and TREM2 was found to aggravate pneumococcal pneumonia and respiratory infection with Sendai virus^43, 75^.

Until now, TREM2’s effects were mainly attributed to its expression in macrophages, monocytes and dendritic cells. Thus, it is interesting that our results indicate that TREM2 expression in non-hematopoietic cells is involved in the observed pathology. TREM2 was not detectable in primary hepatocytes and we, thus, hypothesize that a non-hematopoietic cell population other than hepatocytes is exerting the detrimental effects of TREM2 observed in our model of viral hepatitis. Further studies will need to address the potential involvement of radio-resistant liver-resident cell populations such as hepatic stellate cells and liver sinusoidal endothelial cells. In addition, we cannot rule out neither systemic effects from other cell types outside the liver such as TREM2-expressing adipocytes^76^, nor a role for the soluble form of TREM2^75^.

The seminal study of Wang *et al*. showed that TREM2 binds to phosphatidylcholines and sphingomyelins, assigning a lipid-sensing role to this surface receptor^38^. Interestingly, these are the lipid species we found altered in mice upon infection with LCMV. TREM2 may also sense apoptotic cells through exposed phosphatidylserines^38^. Additional studies implicated ApoE, which plays an important role in lipoprotein metabolism and in immune-related processes and diseases^77–79^, as another ligand for TREM2^34, 35, 37^. *ApoE* ^−/−^ mice infected with LCMV develop exacerbated liver damage^80^, which may suggest the involvement of ApoE in the TREM2-dependent liver pathology. More recently, *Trem1* ^−/−^ mice were reported to be protected from LCMV-induced hepatitis despite normal CD8 T cell responses^81^. TREM1, whose physiological ligands include peptidoglycan recognition protein 1 and likely others^82^, was found to be mostly expressed in neutrophils and suggested to modulate their inflammatory activity. Our bone marrow chimera experiments indicate that TREM2 is unlikely to play a role in neutrophils, which is supported by the notion that TREM1 is typically expressed by activated neutrophils^83, 84^ while TREM2 is not. Thus, the fact that both *Trem2* ^−/−^ and *Trem1* ^−/−^ mice show a protective phenotype in viral hepatitis indicates distinct cell type-specific and non-redundant roles of these TREM family members in liver pathology.

The technological advancements in mass-spectrometry based lipidomics enable to map changes in the composition of circulating lipids during infection. Several studies have reported variations in serum levels of specific sphingolipids in chronic HBV and HCV patients and associated them with disease progression such as fibrosis and hepatic steatosis or antiviral treatment outcome^25, 28–31^. Systematic efforts will be needed to study the biology of individual lipid species in the context of liver pathology and other diseases. It will be crucial to investigate which species are released into the serum by active secretion and which may represent danger-associated molecular patterns from cellular debris at a given time, through which receptors these lipids are sensed by cells and how signal transduction contributes to inflammation. In-depth knowledge about lipid metabolism, perturbations, sensing and signaling *in vivo* will allow to identify disease-relevant dynamic patterns and assign prognostic value. It is understood that LCMV only shares certain features of immunobiology with HBV and HCV^19, 85, 86^, and lipid metabolism is known to differ considerably between mouse and man^87, 88^. Yet, this versatile experimental system may provide valuable contributions to characterize infection-induced global changes in serum metabolites and dissect the affected downstream signaling cascades. Of note, *Trem2* ^−/−^ mice subjected to the concanavalin A-induced acute hepatitis model revealed elevated serum transaminases similar to WT mice, which may indicate that the TREM2-dependent pathology requires context-dependent parameters such as time kinetic, antigen specificity or distinct inflammatory milieus found in viral infections.

Our study revealed global changes in circulating lipids in the sera of LCMV-infected mice and identified a novel detrimental role for the non-hematopoietically expressed lipid-sensor TREM2 in a model of viral hepatitis. This may suggest novel ways to ameliorate virus-induced liver pathologies. Numerous additional functional links between infection-induced changes in serum metabolites and immunoregulation are expected to be uncovered^4^, which will allow novel insights into translational immmunometabolism of inflammatory and infectious diseases.

## Methods

### Mice

*Trem2* ^−/−^ mice^52^ (originally provided by Marco Colonna, Washington University, St. Louis) were back-crossed onto a > 98% C57BL/6 background facilitated by genome-wide SSLP typing at 10 cM intervals (Speed Congenics Facility of the Rheumatic Diseases Core Center). Age matched 8–12 week old *Trem2* ^−/−^ mice and WT C57BL/6 controls were used for all experiments. *CD45*.*1* mice^89^ (obtained from Jackson Laboratories, Bar Harbor, Stock No 002014) were used as controls for bone marrow chimera experiments. All experiments were conducted in individually ventilated cages under specific pathogen-free conditions at the Department for Biomedical Research of the Medical University of Vienna.

For the generation of chimeric mice, bone marrow was harvested by flushing the femurs and tibias of donor mice as previously described^90^. Recipient mice (WT and *Trem2* ^−/−^) were exposed to a single dose of γ-irradiation (9 Gy) and reconstitution of bone marrow cells was achieved by injecting 2 × 10^6^ of the freshly isolated cells from WT or *Trem2* ^−/−^ donor mice. Four weeks later, chimeric mice were treated with 200 μg of anti-CD90 antibody intraperitoneally (i.p.), to deplete any remaining peripheral T cells from the recipient mouse. Another three weeks later, the mice received 100 μl of liposomal clodronate (ClodronateLiposomes.com) i.p., per 10 g of body weight to deplete any remaining macrophages from the recipient. > 4 weeks later mice were used for infection experiments.

### Mouse infections

Mice were infected intravenously (i.v.) with 0.3–1 × 10^6^ focus forming units of LCMV strain WE. LCMV WE was propagated on L929 cells (European Collection of Authenticated Cell Cultures (ECACC) Catalogue No.: 85011425). LCMV titers in serum and organs were determined by immunological focus forming assay as previously described^62^ albeit modified by using Vero cells (originally obtained from Daniel D. Pinschewer, University of Basel).

### Targeted Metabolomics

Targeted metabolomics was performed using the AbsoluteIDQ p180 kit (Biocrates Life Sciences AG, Innsbruck, Austria). The kit allows the identification and quantitation of metabolites by LC- and flow injection analysis (FIA)-MRM. All serum samples were stored at −80 °C until being analyzed on an AB SCIEX QTrap 4000 mass spectrometer using an Agilent 1200 RR HPLC system, which were operated with Analyst 1.6.2 (AB SCIEX). The chromatographic column was obtained from Biocrates. The serum samples and additional blanks, calibration standards and quality controls were prepared according to the user manual. The experiments were validated with the supplied software (MetIDQ, Version 5-4-8-DB100-Boron-2607, Biocrates Life Sciences, Innsbruck, Austria). Quality controls were analyzed every 20^th^ sample. Data was analyzed and visualized with R Studio. For metabolites that were below the limit of detection (LOD), the LOD was used for calculating fold changes and p-values.

### Extraction of serum lipids and pure lipid species

Total lipids were extracted from sera from uninfected mice, and mice infected with LCMV WE eight days previously as follows; Briefly, 100 µl of serum was mixed with 150 µl of 0.5 M KH_2_PO_4_, 750 µl of chloroform and 250 µl of methanol. The mixture was then vortexed for two minutes and then centrifuged for five minutes at 500× g at room temperature. The lower lipid phase was collected and stored at −80 °C, before being used to stimulate the cells 1:20.

The pure lipid species 1,2-dipalmitoleoyl-*sn*-glycero-3-phosphocholine (16:1 (∆9-Cis) PC, Catalog No.: 850358), 1-palmitoyl-2-linoleoyl-*sn*-glycero-3-phosphocholine (16:0–18:2 PC, Catalog No.: 850458 P), N-stearoyl-D-*erythro*-sphingosylphosphorylcholine (18:0 SM (d18:1/18:0), Catalog No.: 860586) and N-palmitoyl-D-*erythro*-sphingosylphosphorylcholine (16:0 SM (d18:1/16:0), Catalog No.: 860584) were purchased from Avanti Polar Lipids and solubilized as previously described^8^. Briefly, lyophilized lipids were reconstituted in ethanol:dodecane (98:2) and sonicated twice for five minutes at room temperature, vortexing in between. The lipids were then stored at −20 °C in glass vials with Teflon lids with an argon gas overlay, before being used to stimulate cells at a concentration of 15 µM.

### Ppar-δ reporter assay

For a dual reporter assay (Promega E1960), MEFs were seeded at 2 × 10^5^cells/ml, were transfected with PPRE luciferase promoter constructs (provided by Nikolina Papac, Medical University of Vienna) and a vector encoding PPAR-δ (provided by Ajay Chawla, University of California, San Francisco) using Lipofectamine LTX (Sigma Aldrich). On the next day, transfected cells were transferred to a 96-well plate at a density of 5 × 10^4^/well and stimulated with either extracted lipids of uninfected or infected WT animals (see above) or pure lipid species (see above). Luciferase activity was measured after 48 h using the Dual-Luciferase Reporter Assay System according to the manufacturer’s instructions (Promega). pRenilla luciferase (Promega) activity was used to control the transfection efficiency. Background was measured in non transfected control cells and subtracted. All obtained luciferase values were normalized to renilla and plotted as fold change to untreated control cells.

### Cell Culture

Primary hepatocyte isolation was conducted as previously described^62^. Bone marrow-derived macrophages (BMDMs) were generated as previously described^43^. Subsequently, cells were infected with LCMV at a MOI of 0.01 and harvested at the respective time points post infection for RNA isolation and real-time PCR analysis.

### RNA isolation and real-time PCR

For measurement of gene expression by real-time PCR analysis, total RNA was isolated using QIAzol lysis reagent according to the manufacturer’s instructions (Qiagen). RNA was then reversed-transcribed into cDNA using the First Strand cDNA synthesis Kit (Fermentas). Subsequent gene expression was analyzed using TaqMan Fast Universal PCR Mastermix and TaqMan Gene Assays (*Trem2*: Mm04209424_g1 and *Gapdh*: 4352339E, Life Tech). Real-time PCR analysis was run on a StepOnePlus Real Time PCR system (Life Technologies). Gene expression was normalized to the housekeeping gene *Eef1a1* ^62^.

### Blood chemistry

Mouse serum was pre-diluted 1:4 or 1:8 in PBS to determine levels of alanine aminotransferase (ALT), aspartate aminotransferase (AST), alkaline phosphatase (AP), bilirubin, blood urea nitrogen (BUN), cholesterol and creatinine using a Cobas C311 Analyzer (Roche).

### Histology

Liver specimens were fixed for 24 hours at room temperature in 4% formaldehyde-PBS solution and paraffin embedded. After cutting, sections (3–4 μm) were deparaffinized using xylene and ethanol, stained with hematoxylin and eosin and a trained pathologist analyzed (blinded) liver pathology using a scoring system, considering lobular and portal inflammation as well as endothelitis and thrombus formation. Each parameter was scored from 0 to 4 points, with 0 representing absent, 1 very mild, 2 mild, 3 moderate and 4 severe. For staining of cleaved caspase 3 and LCMV NP, antigen-retrieval was performed using citrate buffer pH6.0 (Vector laboratories) followed by blocking of endogenous peroxidase with 2% H_2_O_2_ and blocking steps using an avidin biotin blocking kit (Vector laboratories) and rabbit serum. Thereafter sections were incubated over night in either anti-cleaved caspase 3 antibody (Cell signaling) or anti-LCMV nucleoprotein rabbit serum. On the next day, the slides were washed with PBS and incubated with biotinylated swine anti-rabbit IgG (Dako) and developed using the Vectastain ABC kit (Vector laboratories) and DAB-HRP conjugate (Vector laboratories). After counterstaining with hematoxylin and embedding, the sections were analyzed by light microscopy. From each mouse 10 random pictures were taken with a 20-fold magnification and positive cells/field were counted by eye. For the LCMV positive cells, large polyglonal hepatocytes were distinguished from small spindle-shaped infected cells, which were considered as Kupffer cells^61^.

### Flow cytometric analysis of immune cell populations

Single cells suspensions of liver and spleen tissue were prepared by mechanical disruption using a 70 μm cell strainer. Absolute cell numbers were counted with a Neubauer Improved chamber Cells were stimulated with 10^−6^ μM of the respective peptides and subsequently stained with the antibodies CD8b.2 Pacific Blue (clone 54-5.8, Biolegend), B220 APC-eFluor 780 (clone RA3-6B2, eBioscience), IFNγ PE/Cy7 (clone XMG1.2, Biolegend), CD4 FITC (clone H129.19, Biolegend) and TNFα APC (clone MP6-XT22, Biolegend) as previously described^62^.

For testing the reconstitution of cells after bone marrow transplantation, we analyzed blood, liver and spleen of WT (CD45.2) mice that were reconstituted with bone marrow of CD45.1 congenic mice using the following antibodies: CD3 APC (clone 17A2, eBioscience), CD19 FITC (clone eBio1D3, BD), CD11b PerCP Cy5.5 (clone M1/70, eBioscience), CD45.1 Pacific blue (clone A20, Biolegend) and Ly5.2 PE (clone 104, Biolegend). Flow cytometric measurements were performed on a FACS LSR II Fortessa and on FACS Calibur (BD, San Diego, California, United States).

For flow cytometric analysis of different immune cell subsets, livers were cut into small pieces. After incubation in 0.05% collagenase/dispase and 0.01% trypsin inhibitor at 37 °C, liver samples were pressed through a 40 µm cell strainer, centrifuged at 800 g for 5–10 min at 4 °C and resuspended in RPMI 1640. The suspension was then overlayed onto a 33% (vol/vol) Percoll Solution (15 ml) and centrifuged for 30 min at room temperature with no brake. The supernatant was aspirated and red blood cell lysis was performed. After further washing and centrifugation steps, leukocytes were incubated with the following antibodies and analysed by flow cytometry: NK1.1 eF450 (PK136, eBioscience), CD3 V500 (17A2 BioLegend), Ly-6C BV605 (HK1.4, BioLegend), F4/80 APC (BM8, BioLegend), γδTCR PerCP Cy5.5 (GL3 BioLegend), CD19 PE (6D5, BioLegend), CD11b PE Cy7 (M1/70, BD Pharmingen), Ly-6G (1A8, BioLegend), CD11c AF700 (N418, eBioscience).

### *In vivo* Cytotoxicity Assay

Cytotoxic activity of CD8^+^ T cells was measured as previously described^91^. Briefly, WT and *Trem2* ^−/−^ mice were infected with LCMV WE. Eight days post infection syngeneic splenocytes from uninfected C57BL/6 mice were pulsed with the LCMV immune-dominant epitope GP_33-41_ (KAVYNFATC) at a concentration of 10 µM in RPMI 10% FCS with 2-mercaptoethanol for 4 hours. GP_33-41_-pulsed and unpulsed cells were fluorescently labelled (Cell Proliferation Dye eFluor 450, eBioscience) at two different concentrations. After labelling, the two populations were mixed in equal ratios. Virus-infected or uninfected control mice were intravenously injected a total of 1.5 × 10^7 cells. Four hours later, the ratio of eFluor^hi^ (GP_33-41_) and eFlour^low^ (unpulsed) cells was determined in the blood of recipient mice via flow cytometry. Specific killing was calculated as 100 – ([(% eFluor^hi^ infected mouse/% eFluor^low^ infected mouse)/(% eFluor^hi^ uninfected mouse/% eFluor^low^ uninfected mouse)] × 100).

### Concanavalin A-induced acute hepatitis model

Mice were injected intraperitoneally (i.p.) with either concanavalin A (10 mg/kg, Sigma L7647) or PBS as control. Serum was collected at 24 hours post treatment.

### Serum Cytokine Measurements

For quantification of systemic interferon alpha (IFNα), serum was pre-diluted 1:10 and detected by ELISA using rat anti-mIFNα capture antibody (PBL Assay Science 22100-1), rabbit anti-mIFNα detection antibody (PBL Assay Science 32100-1), peroxidase conjugated anti-rabbit secondary antibody (Jackson ImmunoResearch 711-036-152), TMB chromogen solution (Life Technologies 00-2023) and 0.18 M H_2_SO_4_ stopping solution. Absorbance was read at 450 nm using a SpectraMax M5 (Molecular Devices) and used to calculate IFNα concentration as shown previously^62^. Systemic levels of interleukin-6 (eBioscience; 88-7064-88) and CXCL1 (R&D Systems; DY453) were determined using commercial ELISA kits, following the manufacturer’s instructions.

### Ethics Statement

All *in vivo* experiments were performed after approval by the Institutional Review Board of the Medical University of Vienna respectively the Veterinary University of Vienna and the Austrian Ministry of Sciences (permit numbers: BMWF-66.009/0318-II/3b/2012 and BMWFW-68.205/0032-WF/II/3b/2014) and in adherence to the Austrian law for animal experimentation.

### Statistical analyses

Results are shown as line graph or bar graph with the mean + /− the standard error of the mean as indicated. Statistical differences between experimental groups were calculated by either unpaired t-test, One-way ANOVA or Two-way ANOVA with Bonferroni correction as indicated in the respective figure legends. Significant p-values were calculated as follows; *p ≤ 0.05, **p ≤ 0.01, ***p ≤ 0.001 or ****p ≤ 0.0001. Graphs and statistical tests were done with GraphPad Prism 6.

## Electronic supplementary material

Supplementary Information

Table S1
