# Supplementary material for: The lipid-sensor TREM2 aggravates disease in a model of LCMV-induced hepatitis
Source: Sci Rep. 2017 Sep 12;7:11289. doi: 10.1038/s41598-017-10637-y (PMC5595927; doi:10.1038/s41598-017-10637-y)

Supplementary Information

The lipid-sensor TREM2 aggravates disease in a model of LCMV-induced hepatitis

Lindsay Kosack[1,*], Riem Gawish[1,2,3*], Alexander Lercher[1], Bojan Vilagos[1], Anastasiya Hladik[1,2], Karin Lakovits[1,2], Anannya Bhattacharya[1,§], Christopher Schliehe[1,§§], Ildiko Mesteri[4], Sylvia Knapp[1,2,**] and Andreas Bergthaler[1,**]

Fig. S1 Serum lipid changes on day 2 and Ppar-δ reporter assay. (**A**) Serum from WT mice infected with LCMV strain WE two days previously was used for targeted metabolomics (n=4 mice per group). Bubble plot of individual glycerophospholipids (circles) and sphingolipids (triangles) depicting fold-change (x-axis), p-value (y-axis) and the absolute change (size of symbols). Red symbols reflect metabolites with a fold-change >1.5 and a p-value <0.05. (**B-C**) Ppar-δ reporter assay results from WT and *Trem2*$^{-/-}$ mouse embryonic fibroblasts stimulated with serum-extracted lipids (**B**, n=3-4) or pure lipids (**C**, n=4) as described in Methods. Statistical significance was calculated by unpaired t-test. Bars represent the mean ± SEM.

Fig. S2 Similar kidney-specific serum parameters in WT and *Trem2*$^{-/-}$ mice. (**A-B**) WT and *Trem2*$^{-/-}$ mice were infected with LCMV strain WE. Serum levels of blood urea nitrogen (BUN) (**A**) and creatinine (**B**) were measured eight and ten days post infection (n=5 mice per group, representative of two independent experiments). Statistical significance was calculated by Two-way ANOVA with Bonferroni correction. Bars represent the mean ± SEM.

Fig. S3 Similar viral propagation in primary WT and *Trem2*$^{-/-}$ macrophages. BMDMs from WT and *Trem2*$^{-/-}$ mice were isolated *ex vivo* and left uninfected or infected with LCMV strain WE

(MOI 0.01). Viral loads from cell culture supernatants were quantified by immunological focus assay (n=3 mice per group, representative of two independent (similar) experiments). Symbols represent the mean ± SEM.

Fig. S4 CD8$^+$ and CD4$^+$ effector memory T cell responses and FACS gating strategy for intracellular cytokine staining. WT and *Trem2*$^{-/-}$ mice were infected with LCMV strain WE **(A-F)**. CD44^{hi} CD62^{lo} CD8$^+$ effector memory T cells were quantified in liver and spleen **(A-B)** before, four and eight days post infection (n=4 mice per genotype). CD44^{hi} CD62^{lo} CD4$^+$ effector memory T cells were quantified in liver and spleen **(C-D)** before, four and eight days post infection (n=4 mice per group). Statistical significance was calculated by Two-way ANOVA. Bars represent the mean ± SEM. **(E-F)** FACS gating strategy for data shown in Fig. 4.

Fig. S5 Concanavalin A induced acute hepatitis in WT and *Trem2*$^{-/-}$ mice. (**A**) WT and *Trem2*$^{-/-}$ mice received either PBS or 10 mg/kg concanavalin A i.p. **(A-B).** Serum kinetics of ALT (A) and AST (B) were measured 24 hours post treatment (n=6 infected mice and one untreated mouse per genotype). Statistical significance was calculated by unpaired t test. Bars represent the mean ± SEM.

Fig. S6 Profiling of lymphocyte populations in LCMV-infected WT and *Trem2*$^{-/-}$ mice. WT and *Trem2*$^{-/-}$ mice were infected with LCMV strain WE **(A-F)**. γ/δ T cell (CD19$^-$ CD3$^+$ TCRγ/δ$^+$), NK cells (CD19$^-$ CD3$^-$ NK1.1$^+$), NKT cells (CD19$^-$ CD3$^+$ NK1.1$^+$), neutrophils (CD19$^-$ Ly6G$^+$) and macrophages (CD19$^-$ CD11c$^-$ F4/80$^+$) were quantified in liver and spleen before, four and eight days post infection (n=4 mice per group). Statistical significance was calculated by Two-way ANOVA. Bars represent the mean ± SEM.

Fig. S7 Serum cytokine responses in LCMV-infected WT and *Trem2*$^{-/-}$ mice. WT and *Trem2*$^{-/-}$ mice were infected with LCMV WE, and serum was collected at various time points post infection **(A-C)**. Serum kinetics of **(A)** IFNα (12-15 infected and 3 uninfected mice per genotype pooled from three independent experiments), **(B)** interleukin-6 (8-9 mice per genotype pooled from two independent experiments), and **(C)** CXCL1 (4-9 mice per genotype pooled from two independent experiments). Statistical significance was calculated by Two-way ANOVA with Bonferroni correction. Symbols respectively bars represent the mean ± SEM.

Fig. S8 Confirmation of bone marrow reconstitution. Reconstitution of relevant cell types was confirmed by FACS analysis of WT mice that were reconstituted with bone marrow of *CD45.1* mice. Bars represent the mean of 2 mice (**A**). FACS gating strategy (**B**).

Fig. S9 *Trem2* expression in primary macrophages and hepatocytes. (**A**) Bone marrow-derived macrophages and (**B**) primary mouse hepatocytes were seeded at $1x10^6$ cells per well and infected with LCMV at an MOI of 0.01. Expression of *Trem2* and *GAPDH* was analyzed by real-time PCR at the respective time points and normalized to *Eef1a*. Symbols represent the mean ± SEM.

Table S1 Quantification of serum metabolites

Figure S1: Serum lipid changes on day 2 and Ppar-δ reporter assay

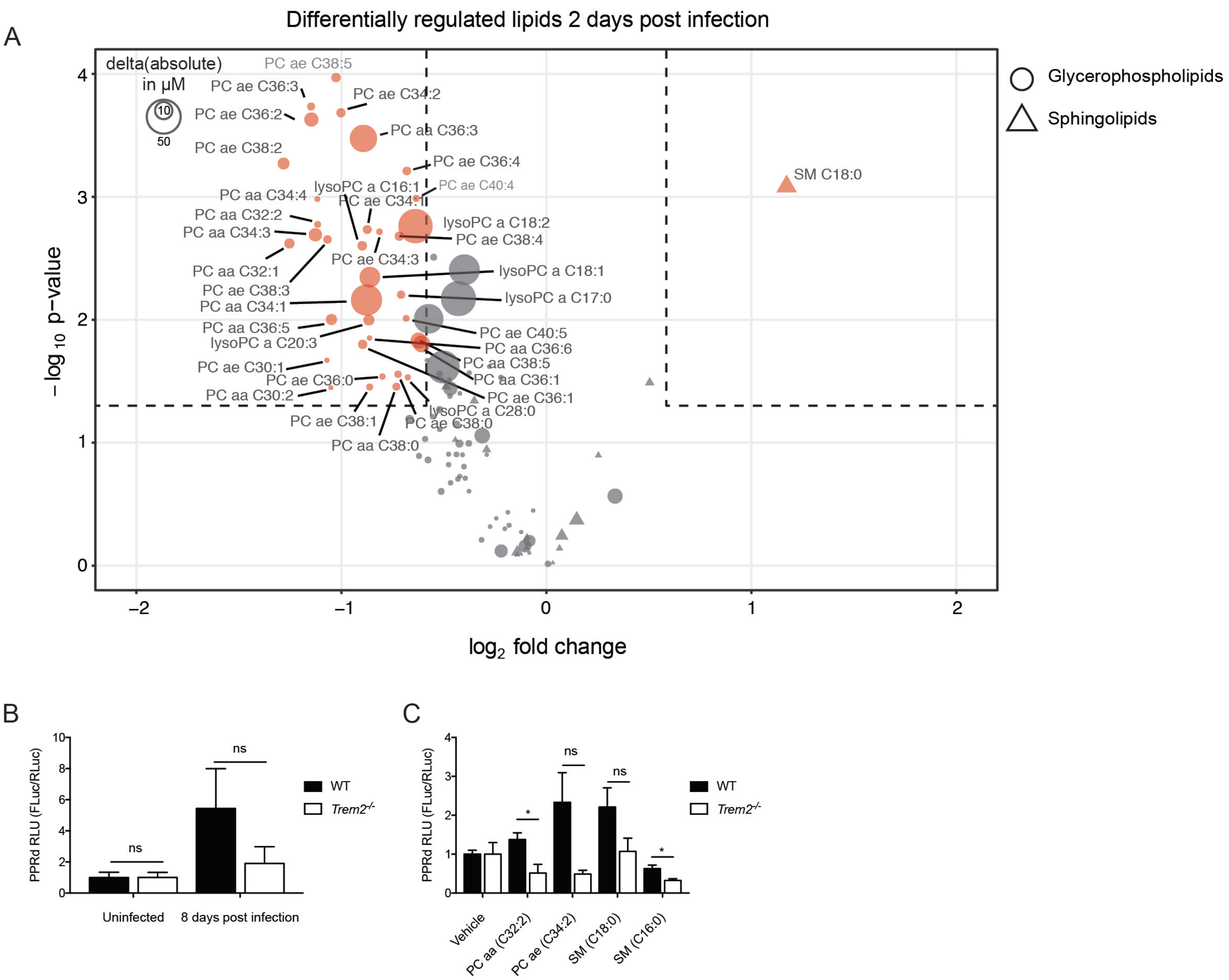

Figure S2: Similar kidney-specific serum parameters in WT and *Trem2*$^{-/-}$ mice

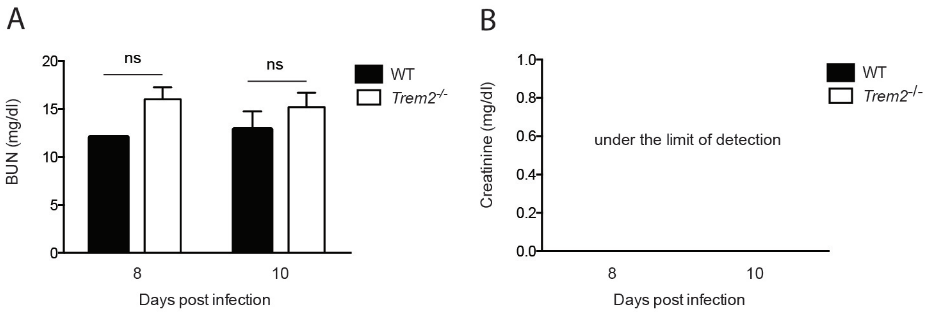

Figure S3: Similar viral propagation in primary WT and *Trem2*$^{-/-}$ macrophages

A

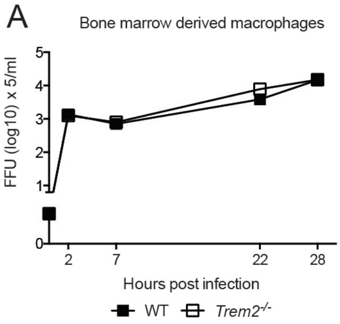

Figure S4: Effector memory T cell responses and FACS gating strategy for intracellular cytokine staining

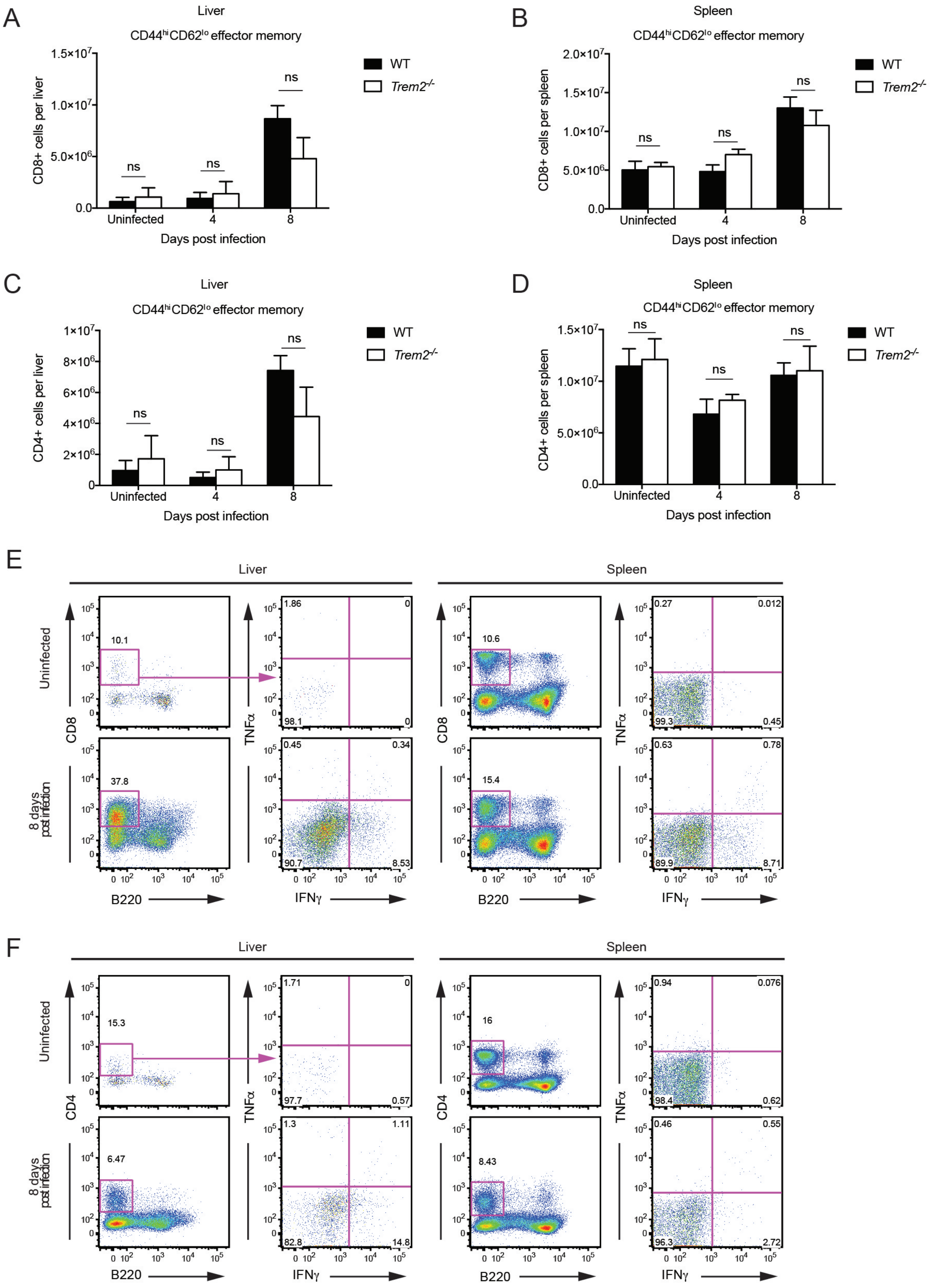

Figure S5: Concanavalin A induced acute hepatitis in WT and *Trem2*$^{-/-}$ mice

A

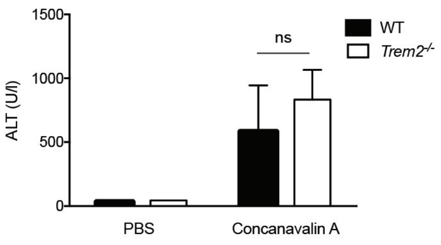

B

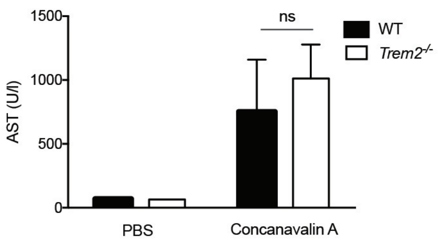

Figure S6: Profiling of lymphocyte populations in LCMV-infected WT and *Trem2*$^{-/-}$ mice

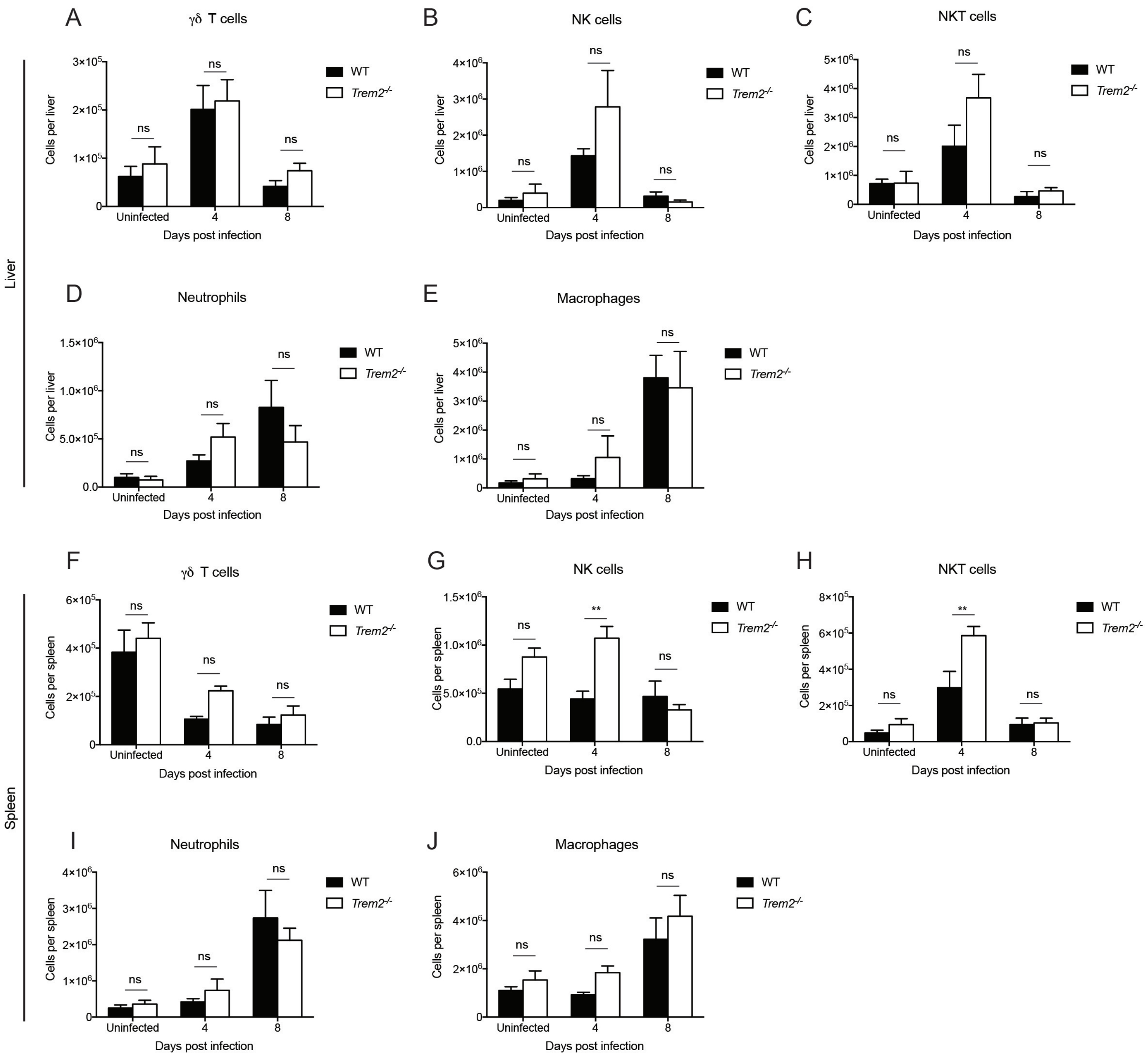

Figure S7: Serum cytokine responses in LCMV-infected WT and *Trem2*$^{-/-}$ mice

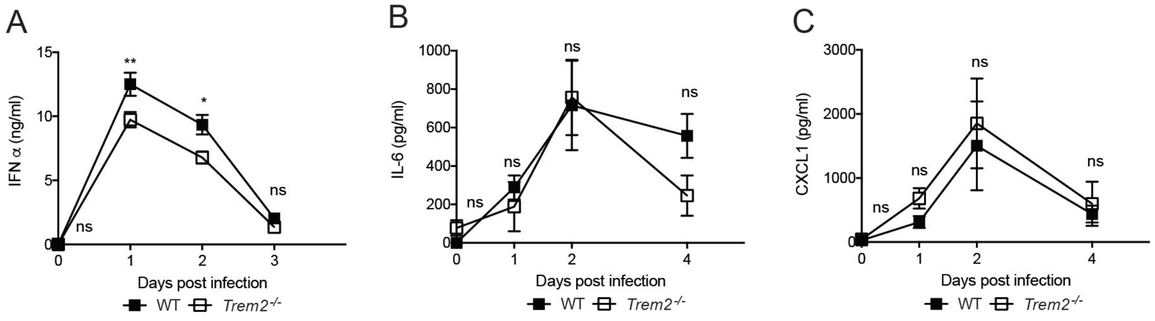

Figure S8: Confirmation of bone marrow reconstitution

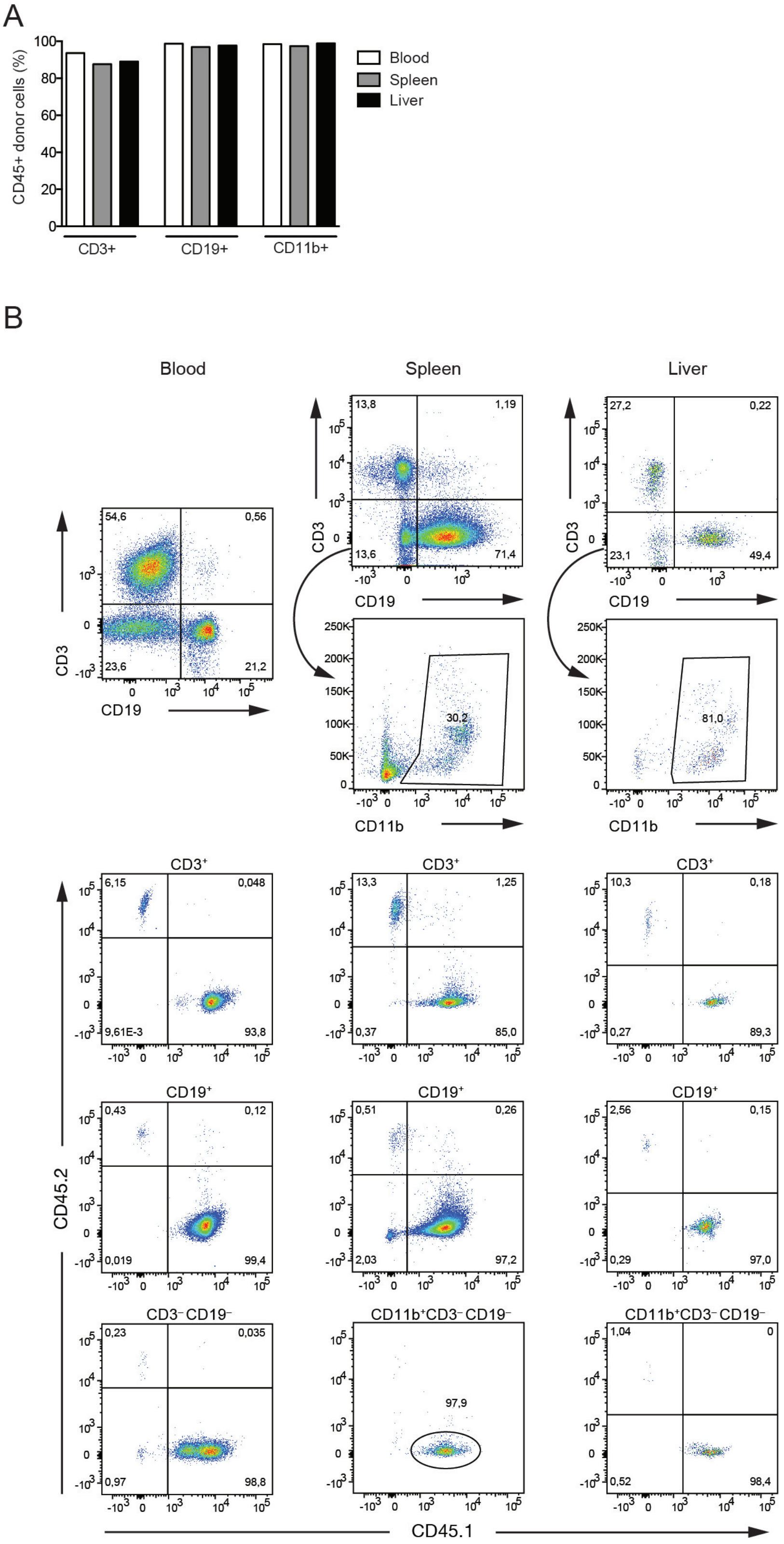

Figure S9: *Trem2* expression in primary mouse hepatocytes

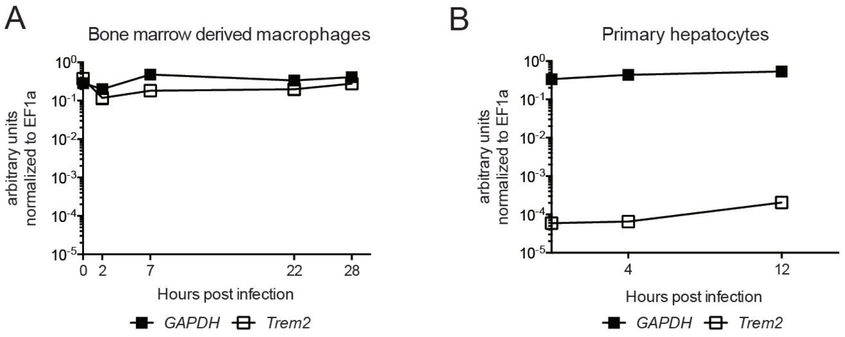

Supplement: Supplementary file 1 — Supplementary Information [file 41598_2017_10637_MOESM1_ESM.pdf]
